# Evaluation of TH17 profile in common variable immunodeficiency patients with or without autoimmunity

**DOI:** 10.1186/1939-4551-8-S1-A236

**Published:** 2015-04-08

**Authors:** Ana Karolina Barreto De Oliveira, Maria Angelica Alcala Neves, Karina Carvalho Salmazi, Joao Miraglia, Myrthes Toledo Barros, Jorge Kalil, Cristina Kokron

**Affiliations:** 1 University of São Paulo Medical School, Brazil; 2 Division of Clinical Immunology and Allergy, Laboratory of Clinical Immunology and Allergy (LIM-60), School of Medicine, University of São Paulo, ZIP CODE: 01246-903, São Paulo, São Paulo, Brazil; 3 Hospital Das Clínicas -Faculdade De Medicina –USP, Brazil; 4 Heart Institute (InCor), Brazil

## Background

Common Variable Immunodeficiency (CVID) is the most common symptomatic primary antibody deficiency in clinical practice and it is characterized by hypogammaglobulinemia, increased susceptibility to infections, autoimmune diseases and malignancies. The pathogenesis of CVID is still not well established. Immunologic abnormalities found in CVID patients include defects in B cell differentiation, in T cell function, altered receptor expression and cytokine production, regulatory cell disturbances and disorders of innate immunity. The aim of this study was to evaluate the T_H_17 profile of CVID patients, in order to observe if CVID patients with autoimmunity present a T_H_17 polarized cellular response pattern.

## Methods

Forty two CVID patients were recruited from the PID outpatient clinic, Clinical Immunology and Allergy Division of HC-FMUSP, being 17 with and 25 without autoimmunity. T lymphocyte characterization was done by flow cytometry using the following panels: activation panel (CD3, CD4, CD8, HLA-DR, CD38, CD69, *Live/dead*); regulatory T cells panel (CD3, CD4, CD8, CD25, CD39, CD45-RO, CD127, *Live/dead*, FoxP3); and functional panel - upon 5 hours stimulation - (CD3, CD4, CD8, *Live/dead*, TNF-α, IFN-γ_,_ IL-2, IL-17, IL-21).

## Results

No difference was found in the T_H_17 profile (% CD4^+^IL-17^+^ cells) between CVID patients with/without autoimmunity. Likewise, T_H_17 profile was not different when all CVID patients were compared to controls. We observed a reduction in Treg frequency in CVID patients with autoimmunity in comparison to patients without autoimmunity as well as controls. CVID patients presented increased expression of activation markers in CD4 and CD8 T cells when compared to controls. Finally, increased percentage of IL-17 producing CD8 T cells (Tc17) was observed in patients with autoimmunity.

## Conclusions

We conclude that in CVID, T_H_17 cells may not be responsible for the induction of autoimmunity which is possibly consequent to the several immune dysregulations found in this immunodeficiency. More studies are necessary to establish T_H_17 and Tc17 function in CVID pathogenesis.

